# COVIDNet-CT: A Tailored Deep Convolutional Neural Network Design for Detection of COVID-19 Cases From Chest CT Images

**DOI:** 10.3389/fmed.2020.608525

**Published:** 2020-12-23

**Authors:** Hayden Gunraj, Linda Wang, Alexander Wong

**Affiliations:** ^1^Department of Mechanical and Mechatronics Engineering, University of Waterloo, Waterloo, ON, Canada; ^2^Vision and Image Processing Research Group, University of Waterloo, Waterloo, ON, Canada; ^3^DarwinAI Corp., Waterloo, ON, Canada; ^4^Waterloo Artificial Intelligence Institute, University of Waterloo, Waterloo, ON, Canada

**Keywords:** COVID-19, computed tomography, deep learning, image classification, SARS-CoV-2, pneumonia

## Abstract

The coronavirus disease 2019 (COVID-19) pandemic continues to have a tremendous impact on patients and healthcare systems around the world. In the fight against this novel disease, there is a pressing need for rapid and effective screening tools to identify patients infected with COVID-19, and to this end CT imaging has been proposed as one of the key screening methods which may be used as a complement to RT-PCR testing, particularly in situations where patients undergo routine CT scans for non-COVID-19 related reasons, patients have worsening respiratory status or developing complications that require expedited care, or patients are suspected to be COVID-19-positive but have negative RT-PCR test results. Early studies on CT-based screening have reported abnormalities in chest CT images which are characteristic of COVID-19 infection, but these abnormalities may be difficult to distinguish from abnormalities caused by other lung conditions. Motivated by this, in this study we introduce COVIDNet-CT, a deep convolutional neural network architecture that is tailored for detection of COVID-19 cases from chest CT images via a machine-driven design exploration approach. Additionally, we introduce COVIDx-CT, a benchmark CT image dataset derived from CT imaging data collected by the China National Center for Bioinformation comprising 104,009 images across 1,489 patient cases. Furthermore, in the interest of reliability and transparency, we leverage an explainability-driven performance validation strategy to investigate the decision-making behavior of COVIDNet-CT, and in doing so ensure that COVIDNet-CT makes predictions based on relevant indicators in CT images. Both COVIDNet-CT and the COVIDx-CT dataset are available to the general public in an open-source and open access manner as part of the COVID-Net initiative. While COVIDNet-CT is not yet a production-ready screening solution, we hope that releasing the model and dataset will encourage researchers, clinicians, and citizen data scientists alike to leverage and build upon them.

## 1. Introduction

Coronavirus disease 2019 (COVID-19), caused by severe acute respiratory syndrome coronavirus 2 (SARS-CoV-2), continues to have a tremendous impact on patients and healthcare systems around the world. In the fight against this novel disease, there is a pressing need for fast and effective screening tools to identify patients infected with COVID-19 in order to ensure timely isolation and treatment. Currently, reverse transcription polymerase chain reaction (RT-PCR) testing is the primary means of screening for COVID-19, as it can detect SARS-CoV-2 ribonucleic acid (RNA) in sputum samples collected from the upper respiratory tract (1). While RT-PCR testing for COVID-19 is highly specific, its sensitivity is variable depending on sampling method and time since onset of symptoms (2–4), and some studies have reported relatively low COVID-19 sensitivity (3, 5). Moreover, RT-PCR testing is a time-consuming process which is in high demand, leading to possible delays in obtaining test results.

Chest computed tomography (CT) imaging has been proposed as an alternative screening tool for COVID-19 infection due to its high sensitivity, and may be particularly effective when used as a complement to RT-PCR testing (4–6). CT imaging saw extensive use during the early stages of the COVID-19 pandemic, particularly in Asia. While cost and resource constraints limit routine CT screening specifically for COVID-19 detection (7), CT imaging can be especially useful as a screening tool in situations where:

- Patients are undergoing routine CT examinations for non-COVID-19 related reasons. For example, CT examinations may be conducted for routine cancer screening, monitoring for elective surgical procedures (8), and neurological examinations (9). Since such CT examinations are being conducted as a routine procedure regardless of COVID-19, there are no additional cost or resource constraints associated with leveraging such examinations for COVID-19 screening as well.
- Patients have worsening respiratory status or developing complications that require expedited care (10). In such scenarios, immediate treatment of patients may be necessary and thus CT imaging is conducted on the patient for COVID-19 infection while waiting for RT-PCR testing to confirm COVID-19 infection.
- Patients are suspected to be COVID-19-positive but their RT-PCR tests are negative. For example, patients who have had close contact with confirmed COVID-19 cases and are exhibiting symptoms of the disease are highly suspect, but may have negative RT-PCR results. In these cases, CT imaging may be used to confirm COVID-19 infection pending positive RT-PCR results.

**Figure 1:**
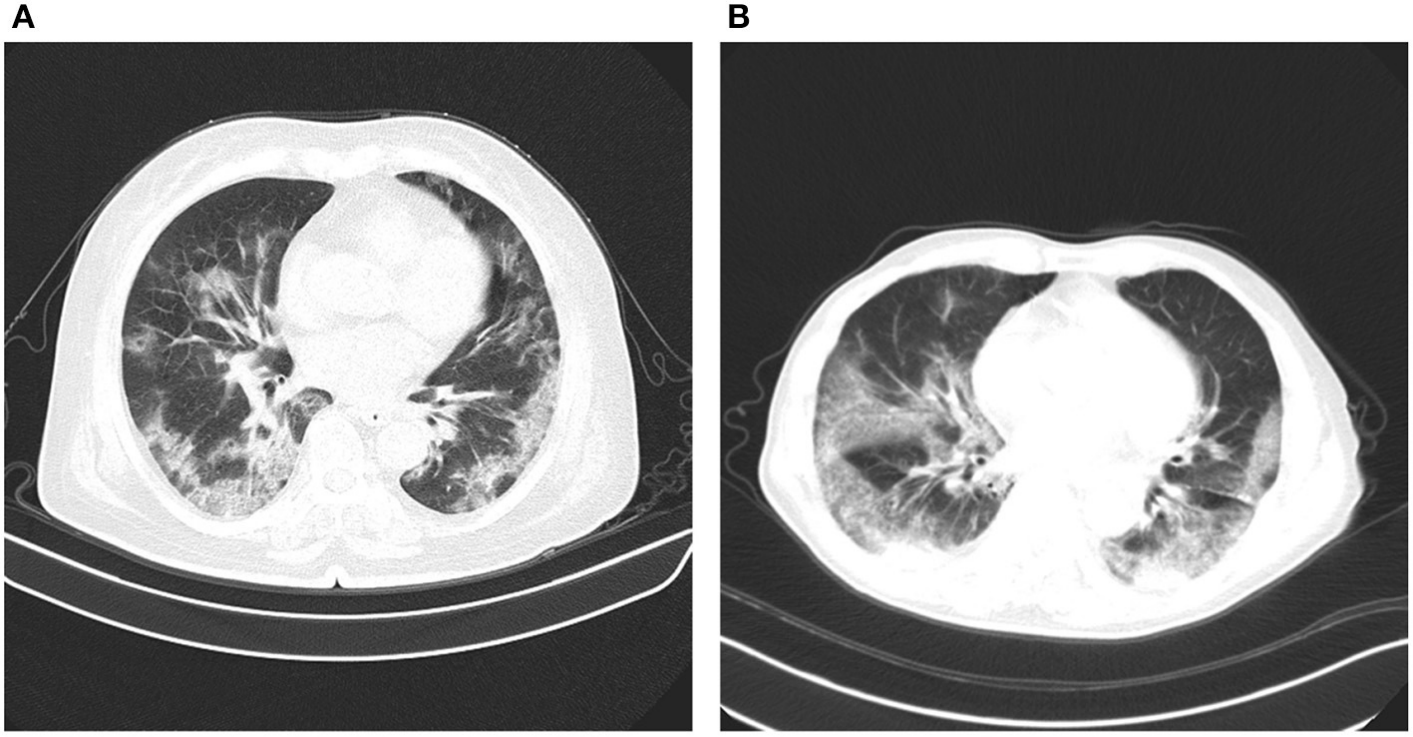
Example chest CT abnormalities in **(A)** a patient with COVID-19 pneumonia, and **(B)** a patient with non-COVID-19 pneumonia. It can be observed that visual differences in abnormalities between COVID-19 pneumonia and non-COVID-19 penumonia can be quite subtle.

In early studies, it was found that certain abnormalities in chest CT images are indicative of COVID-19 infection, with ground-glass opacities, patchy shadows, crazy-paving pattern, and consolidation being some of the most commonly reported abnormalities, typically with bilateral involvement (4–6, 11–14). Moreover, some studies have found that abnormalities in a patient's chest CT scan due to COVID-19 infection may be present despite a negative RT-PCR test (4–6). However, as illustrated in Figure 1, these imaging abnormalities may not be specific to COVID-19 infection, and the visual differences between COVID-19-related abnormalities and other abnormalities can be quite subtle. As a result, the performance of radiologists in distinguishing COVID-19-related abnormalities from abnormalities of other etiology may vary considerably (15, 16). For radiologists, visual analysis of CT scans is also a time-consuming manual task, particularly when patient volume is high or in large studies.

In this study we introduce COVIDNet-CT, a deep convolutional neural network architecture tailored specifically for detection of COVID-19 cases from chest CT images via a machine-driven design exploration approach. While COVID-19 detection from chest CT images has been investigated extensively in previous studies, to the best of the authors' knowledge COVIDNet-CT is the first deep neural network architecture to be built specifically for this task using a machine-driven design exploration strategy, resulting in a highly efficient yet highly accurate deep neural network architecture. Additionally, we describe the creation of COVIDx-CT, a benchmark CT image dataset derived from CT imaging data collected by the China National Center for Bioinformation (CNCB) (17) comprising 104,009 images across 1,489 patient cases. Though this imaging data is not novel, we have cleaned the data and provided additional annotations to allow for others to compare COVID-19 detection methods using a common dataset. Finally, to investigate the decision-making behavior of COVIDNet-CT, we perform an explainability-driven performance validation and analysis of its predictions, allowing us to explore the critical visual factors associated with COVID-19 infection while also auditing COVIDNet-CT to ensure that its decisions are based on relevant CT image features. To the best of the authors' knowledge, this is the first study to leverage GSInquire (18) for this task. In an effort to encourage continued research and development, COVIDNet-CT and the COVIDx-CT dataset are available to the general public^1^ in an open-source and open access manner as part of the COVID-Net (19, 20) initiative, a global open initiative for accelerating collaborative advancement of artificial intelligence for assisting in the fight against the COVID-19 pandemic.

## 2. Materials and Methods

### 2.1. Ethics

This study was reviewed and approved by the University of Waterloo Ethics Board (42235). Written informed consent from the participants or their legal guardian/next of kin was not required to participate in this study in accordance with national legislation and institutional requirements.

### 2.2. COVIDx-CT Dataset

To build the proposed COVIDNet-CT, we constructed a dataset of 104,009 chest CT slices across 1,489 patient cases, which we refer to as COVIDx-CT. Notably, this CT imaging data is not novel, as it is derived from CT imaging data collected by the CNCB (17). Our contribution consists of cleaning and preparing the raw data in a format suitable for benchmarking, as well as providing bounding box annotations for the body regions within the CT images.

**Figure 2:**
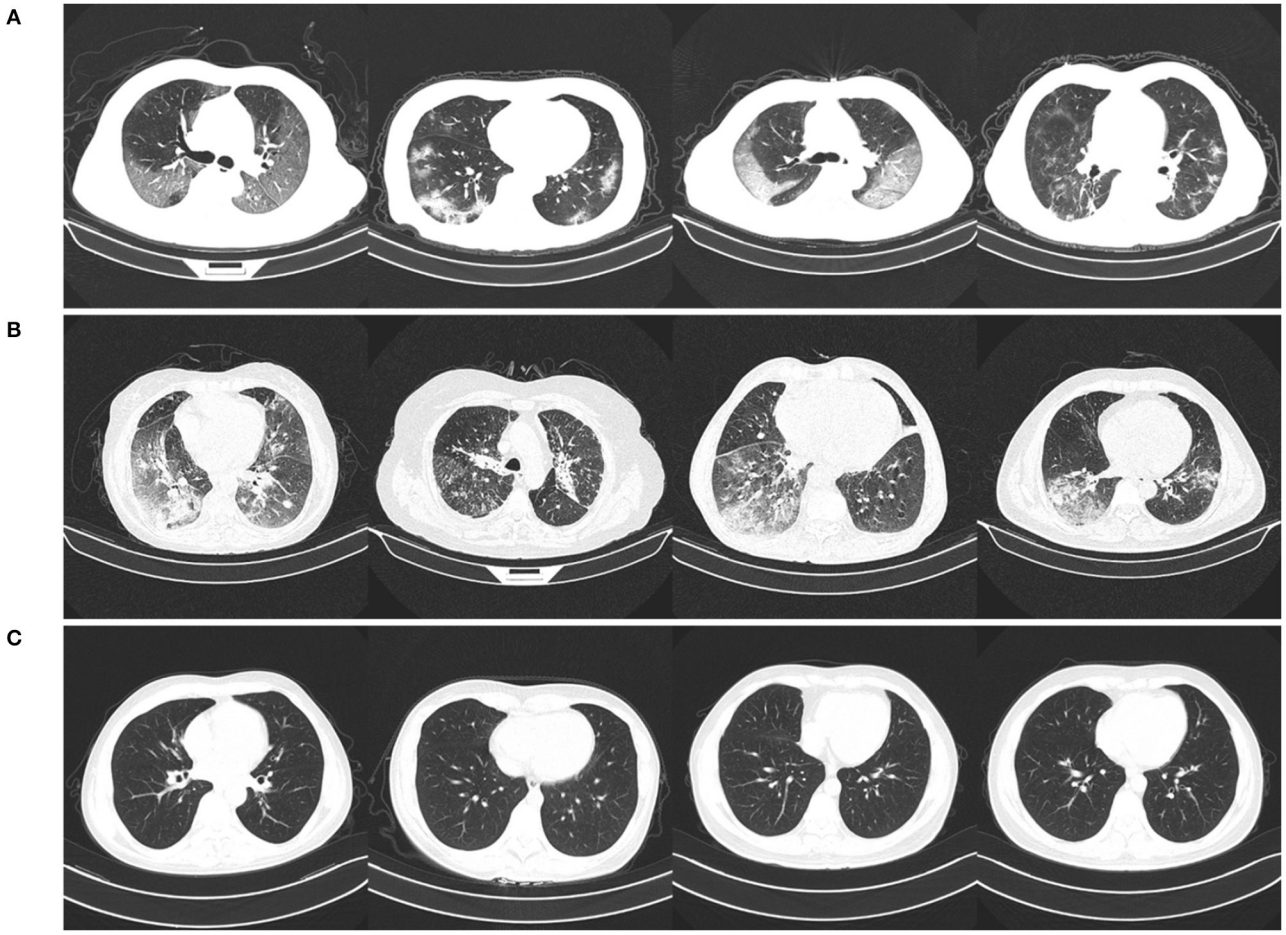
Example chest CT images from the COVIDx-CT dataset, illustrating **(A)** COVID-19 pneumonia cases, **(B)** non-COVID-19 pneumonia cases, and **(C)** normal control cases.

**Figure 3:**
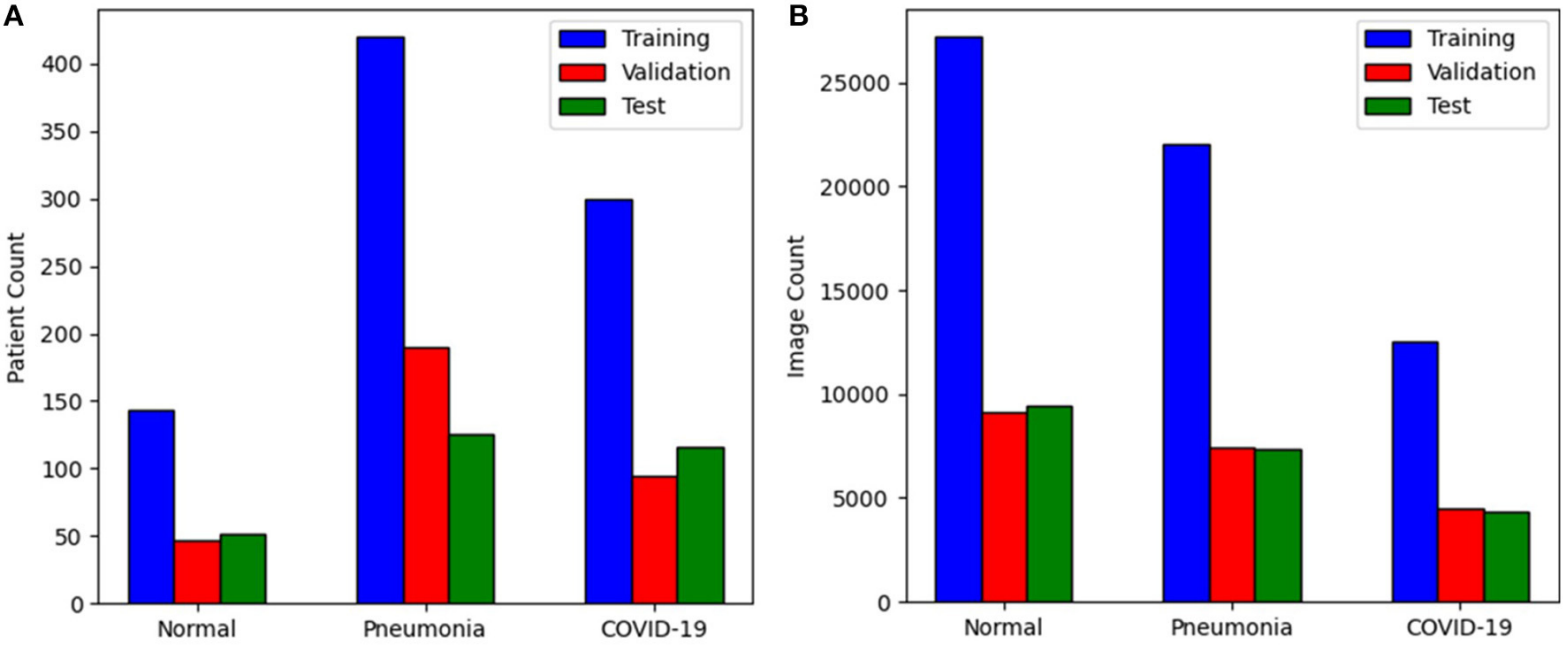
Distribution of the COVIDx-CT dataset amongst training, validation, and test sets by **(A)** patient count and **(B)** image count.

The CNCB data is comprised of chest CT examinations from different hospital cohorts across China as part of the China Consortium of Chest CT Image Investigation (CC-CCII). More specifically, the CT imaging data consists of chest CT volumes across three different infection types: novel coronavirus pneumonia due to SARS-CoV-2 viral infection (NCP), common pneumonia due to non-COVID-19 infections (CP), and normal controls. Figure 2 shows example CT images for each of the infection types from the constructed COVIDx-CT dataset. For NCP and CP CT volumes, slices marked as containing lung abnormalities were leveraged. Additionally, we excluded CT volumes where the background had been removed to leave segmented lung regions, as the contrast present in these images can lead to model biases. To standardize the field-of-view in the CT images, an automatic cropping algorithm was developed to crop the images to the body region. Finally, we split the COVIDx-CT dataset into training, validation, and test sets, using an ~60–20–20% split for training, validation, and test, respectively. These sets were constructed such that each patient belongs to a single set. Figure 3 shows the distribution of patient cases and images in the COVIDx-CT dataset amongst the different infection types and dataset splits.

### 2.3. Machine-Driven Design Exploration

Inspired by Wang and Wong (19), a machine-driven design exploration strategy was leveraged to create the proposed COVIDNet-CT. More specifically, machine-driven design exploration involves the automatic exploration of possible network architecture designs and identifies the optimal microarchitecture and macroarchitecture patterns with which to build the deep neural network. As discussed in Wang and Wong (19), the use of machine-driven design exploration allows for greater flexibility and granularity in the design process as compared to manual design, and ensures that the resulting network satisfies the given operational requirements. As such, a machine-driven design exploration approach would enable the creation of a tailored deep convolutional neural network catered specifically for the purpose of COVID-19 detection from chest CT images in a way that satisfies sensitivity and positive predictive value (PPV) requirements, while also minimizing computational and architectural complexity to enable widespread adoption in clinical environments where computing resources may be limited.

More specifically, in this study we leverage the concept of generative synthesis (21, 22) as our machine-driven design exploration strategy, where the problem of identifying a tailored deep neural network architecture for the task and data at hand is formulated as a constrained optimization problem based on a universal performance function U [e.g., (23)] and a set of quantitative constraints based on operational requirements related to the task and data at hand. This constrained optimization problem is then solved via an iterative strategy, initialized with the data at hand, an initial network design prototype, and the set of quantitative constraints. Here, we specify two key operational requirements as quantitative constraints during the machine-driven design exploration process: (i) COVID-19 sensitivity ≥95% on the COVIDx-CT validation dataset, and (ii) COVID-19 PPV ≥95% on the COVIDx-CT validation dataset. Sensitivity and PPV are calculated using Equations (1) and (3), respectively, which are given in section 3.1. These operational requirements were specified in order to ensure low false-negative and false-positive rates, respectively. For the initial network design prototype, we leveraged residual architecture design principles (24, 25), as they have been shown to enable reliable deep architectures which are easier to train to high performance. Furthermore, the output of the initial network design prototype is a softmax layer corresponding to the following prediction categories: (i) no infection (normal), (ii) non-COVID-19 pneumonia, and (iii) COVID-19 viral pneumonia.

### 2.4. Network Architecture

**Figure 4:**
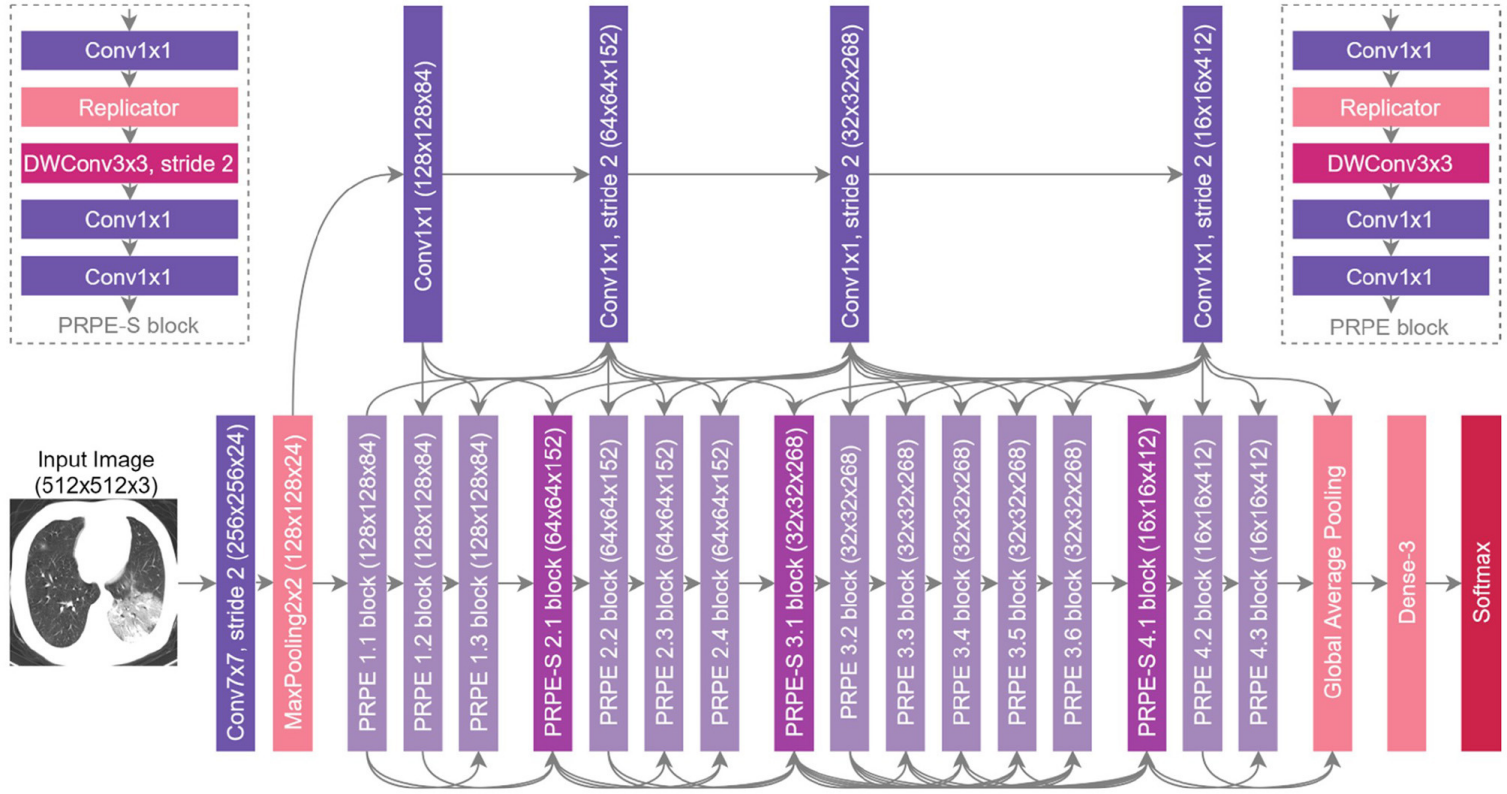
The proposed COVIDNet-CT architecture design via machine-driven design exploration. Notable characteristics include high architectural diversity, selective long-range connectivity, and lightweight design patterns (e.g., PRPE and PRPE-S patterns).

The proposed COVIDNet-CT architecture is shown in Figure 4, and is publicly available at https://github.com/haydengunraj/COVIDNet-CT. As can be seen, the network produced via a machine-driven design exploration strategy exhibits high architectural diversity as evidenced by the heterogeneous composition of conventional spatial convolution layers, pointwise convolutional layers, and depthwise convolution layers in a way that strikes a balance between accuracy and architectural and computational complexity. Further evidence of the high architectural diversity of COVIDNet-CT is the large microarchitecture design variances within each layer of the network (as seen by the tensor configurations of the individual layers shown in Figure 4). Furthermore, the machine-driven design exploration strategy made heavy use of unstrided and strided projection-replication-projection-expansion design patterns, which we denote as PRPE and PRPE-S for unstrided and strided patterns, respectively. These patterns consist of:

1. A projection to lower channel dimensionality via pointwise convolutions.
2. A replication of the projections to increase channel dimensionality efficiently.
3. An efficient spatial feature representation via depthwise convolutions (unstrided and strided for PRPE and PRPE-S, respectively).
4. An expansion of channel dimensionality conducted by pointwise convolutions.

The use of lightweight design patterns, such as PRPE and PRPE-S enables COVIDNet-CT to achieve high computational efficiency while maintaining high representational capacity. While these design patterns may be difficult and time-consuming to design manually, machine-driven design allows for these fine-grained design patterns to be rapidly and automatically discovered. Finally, selective long-range connectivity can be observed, which enables greater representational capabilities in a more efficient manner than densely-connected deep neural networks.

### 2.5. Implementation Details

**Figure 5:**
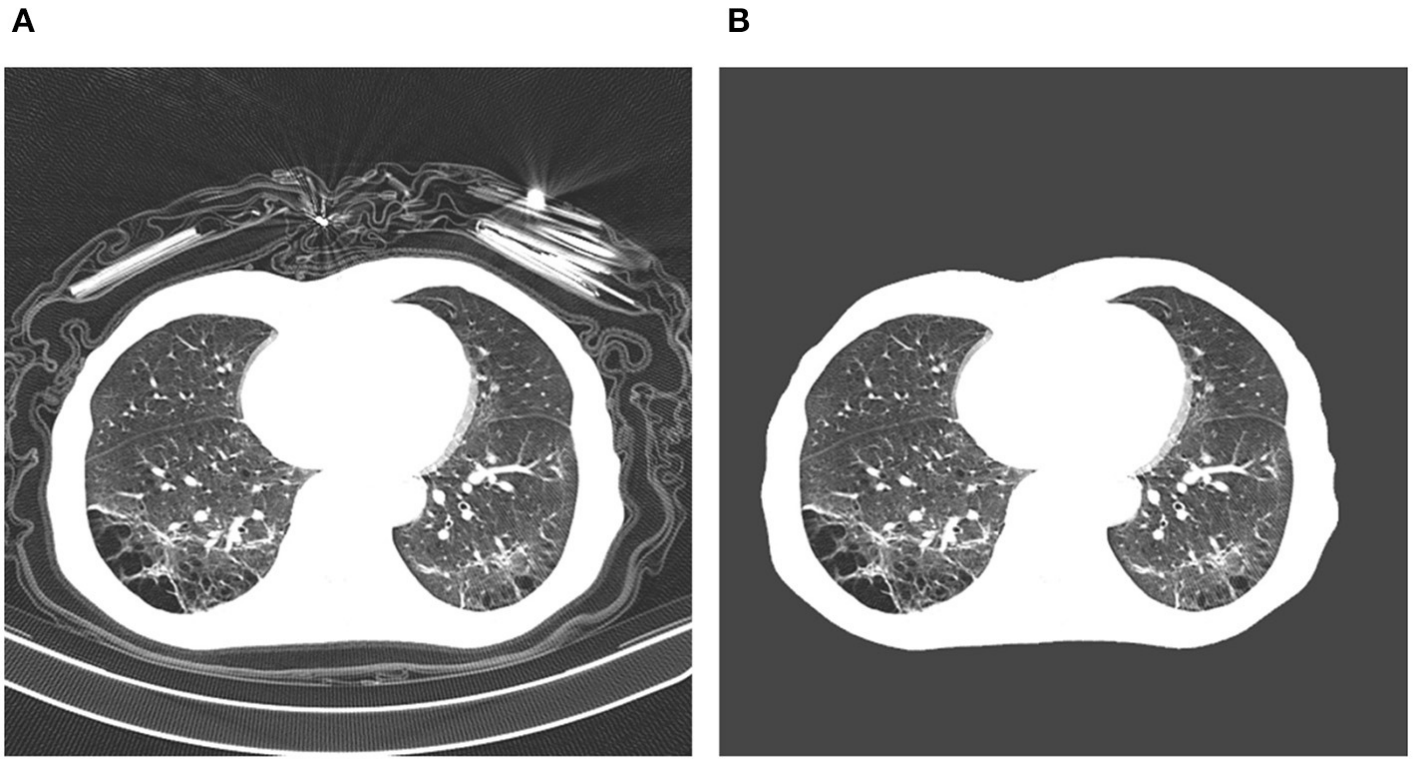
Example COVID-19 case before and after removal of irrelevant visual indicators as part of data augmentation. In **(A)**, a number of irrelevant visual indicators are present, such as the patient table of the CT scanner as well as imaging artifacts. After removing these irrelevant indicators, the image in **(B)** is obtained.

The proposed COVIDNet-CT was pre-trained on the ImageNet (26) dataset and then trained on the COVIDx-CT dataset via stochastic gradient descent with momentum (27). The hyperparameters used for training are as follows: learning rate = 5e-3, momentum = 0.9, number of epochs = 17, batch size = 8. Data augmentation was applied with the following augmentation types: cropping box jitter, rotation, horizontal and vertical shear, horizontal flip, and intensity shift and scaling. In initial experiments, it was found via explainability-driven performance validation (see section 2.6 for more details on the methodology) that erroneous indicators in the CT images (e.g., patient tables of the CT scanners, imaging artifacts, etc.) were being leveraged by the network to make predictions. To help prevent this behavior, we introduce an additional augmentation which removes any visual indicators which lie outside of the patient's body, as illustrated in Figure 5. Finally, we adopt a batch re-balancing strategy similar to that employed in Wang and Wong (19) to ensure a balanced distribution of each infection type at the batch level. The proposed COVIDNet-CT was implemented, trained, and evaluated using the TensorFlow deep learning library (28) and a single NVIDIA Tesla V100 GPU.

### 2.6. Explainability-Driven Performance Validation of COVIDNet-CT

While scalar performance metrics are a valuable quantitative method for evaluating deep neural networks, they are incapable of explaining a network's decision-making behavior. In clinical applications, the ability to understand how a deep neural network makes decisions is critical, as these decisions may ultimately affect the health of patients. Motivated by this, we audit COVIDNet-CT via an explainability-driven performance analysis strategy in order to better understand which CT imaging features are critical to its detection decisions. Moreover, by leveraging explainability, we can ensure that COVIDNet-CT is making decisions based on relevant information in CT images rather than erroneously basing its decisions on irrelevant factors (as we have seen in initial experiments as described in section 2.5). In this study, we leverage GSInquire (18) as the explainability method of choice for explainability-driven performance validation to visualize critical factors in CT images. GSInquire leverages the generative synthesis strategy (21, 22) that was employed for machine-driven design exploration, and was previously shown quantitatively to provide explanations that better reflect the decision-making process of deep neural networks when compared to other state-of-the-art explainability methods (18).

In particular, generative synthesis leverages the interplay between a generator-inquisitor pair {G,I} which work in tandem to obtain improved insights about deep neural networks and generate efficient networks (22). GSInquire in turn leverages the inquisitor I from this process to identify and visualize the critical factors that a network uses to make predictions (18). Unlike approaches that generate heatmaps pertaining to importance variations within an image, GSInquire can identify specific critical factors within an image that have the greatest impact on the decision-making process.

## 3. Results

### 3.1. Quantitative Results

We quantitatively evaluate the performance of the proposed COVIDNet-CT on the COVIDx-CT dataset. For this dataset, we computed the test accuracy as well as sensitivity, specificity, PPV, and negative predictive value (NPV) for each infection type at the image level. These metrics are computed using Equations (1)–(4), respectively. For a particular infection type, TP refers to the number of true-positive predictions, FP refers to the number of false-positive predictions, TN refers to the number of true-negative predictions, and FN refers to the number of false-negative predictions:

(1) $$\begin{array}{l} \text{sensitivity}=\frac{\text{TP}}{\text{TP + FN}} \end{array}$$

(2) $$\begin{array}{l} \text{specificity}=\frac{\text{TN}}{\text{TN + FP}} \end{array}$$

(3) $$\begin{array}{l} \text{PPV}=\frac{\text{TP}}{\text{TP}+\text{FP}} \end{array}$$

(4) $$\begin{array}{l} \text{NPV}=\frac{\text{TN}}{\text{TN + FN}} \end{array}$$

The test accuracy, architectural complexity (in terms of number of parameters), and computational complexity [in terms of number of floating-point operations (FLOPs)] of COVIDNet-CT are shown in Table 1. As shown, COVIDNet-CT achieves a relatively high test accuracy of 99.1% while having relatively low architectural and computational complexity. This highlights one of the benefits of leveraging machine-driven design exploration for identifying the optimal macroarchitecture and microarchitecture designs for building a deep neural network architecture tailored for the task and data at hand. In the case of COVIDNet-CT, the result is a highly accurate yet highly efficient deep neural network architecture that is suitable for scenarios where computational resources are a limiting factor. In clinical scenarios, such architectures may also be suitable for use in embedded devices.

**Table 1:** Comparison of parameters, FLOPs, and accuracy (image-level) for tested network architectures on the COVIDx-CT dataset.

**Architecture**	**Parameters (M)**	**FLOPs (G)**	**Accuracy (%)**
ResNet-50 (25)	23.55	42.72	98.7
NASNet-A-Mobile (29)	4.29	5.94	98.6
EfficientNet-B0 (30)	4.05	**4.07**	98.3
COVIDNet-CT	**1.40**	4.18	**99.1**

*Best results highlighted in bold*.

We next examine the sensitivity, specificity, PPV, and NPV for COVID-19 images, as well as how these statistics could impact the efficacy of COVIDNet-CT in a clinical environment. In Table 2, we observe that COVIDNet-CT achieves a high COVID-19 sensitivity of 97.3%, which ensures that a low proportion of COVID-19 cases are incorrectly classified as non-COVID-19 pneumonia or normal cases. Moreover, given that RT-PCR testing is highly specific, we want to ensure that COVIDNet-CT has high sensitivity in order to effectively complement RT-PCR testing. Next, in Table 3, we observe that COVIDNet-CT also achieves a high COVID-19 PPV of 99.7%, thereby ensuring a low proportion of false-positive predictions which could cause an unnecessary burden on the healthcare system in the form of isolation, testing, and treatment. Finally, we consider COVIDNet-CT's specificity and NPV scores for COVID-19 images as shown in Tables 4, 5, respectively. We observe high values for both of these metrics (99.9% COVID-19 specificity, 99.3% COVID-19 NPV), meaning that negative predictions for COVID-19 are true negatives in the vast majority of cases. This is a useful characteristic in clinical scenarios since it allows for rapid identification of patients who do not have COVID-19.

**Table 2:** Sensitivity for each infection type at the image level on the COVIDx-CT dataset.

**Architecture**	**Normal**	**Non-COVID-19 pneumonia**	**COVID-19**
**Sensitivity (%)**			
ResNet-50 (25)	99.9	98.7	96.2
NASNet-A-Mobile (29)	99.9	97.9	96.8
EfficientNet-B0 (30)	99.8	97.8	95.8
COVIDNet-CT	**100.0**	**99.0**	**97.3**

*Best results highlighted in bold*.

**Table 3:** Positive predictive value (PPV) for each infection type at the image level on the COVIDx-CT dataset.

**Architecture**	**Normal**	**Non-COVID-19 pneumonia**	**COVID-19**
**PPV (%)**			
ResNet-50 (25)	99.3	97.8	99.1
NASNet-A-Mobile (29)	**99.6**	98.2	97.1
EfficientNet-B0 (30)	98.7	97.6	98.6
COVIDNet-CT	99.4	**98.4**	**99.7**

*Best results highlighted in bold*.

**Table 4:** Specificity for each infection type at the image level on the COVIDx-CT dataset.

**Architecture**	**Normal**	**Non-COVID-19 pneumonia**	**COVID-19**
**Specificity (%)**			
ResNet-50 (25)	99.5	98.8	99.8
NASNet-A-Mobile (29)	**99.6**	99.0	99.3
EfficientNet-B0 (30)	98.9	98.7	99.6
COVIDNet-CT	99.5	**99.2**	**99.9**

*Best results highlighted in bold*.

**Table 5:** Negative predictive value (NPV) for each infection type at the image level on the COVIDx-CT dataset.

**Architecture**	**Normal**	**Non-COVID-19 pneumonia**	**COVID-19**
**NPV (%)**			
ResNet-50 (25)	99.9	99.3	99.0
NASNet-A-Mobile (29)	99.9	98.9	99.2
EfficientNet-B0 (30)	99.8	98.8	98.9
COVIDNet-CT	**100.0**	**99.5**	**99.3**

*Best results highlighted in bold*.

**Figure 6:**
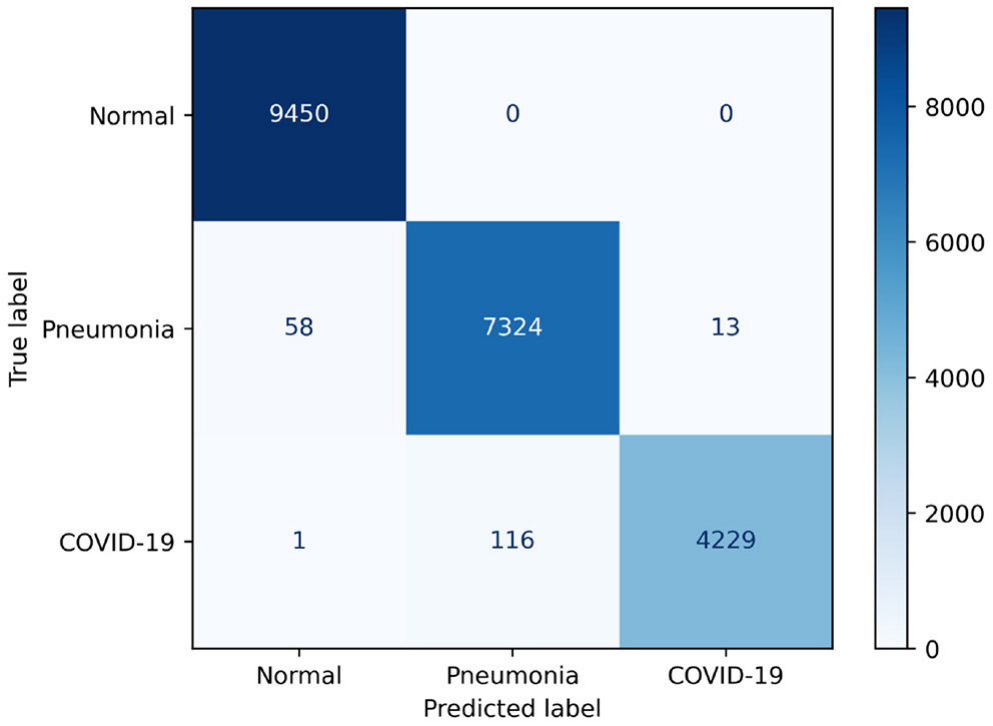
Confusion matrix for COVIDNet-CT on the COVIDx-CT test dataset.

Examining Figure 6, we observe that COVIDNet-CT is extremely effective at distinguishing normal control cases from both COVID-19 and non-COVID-19 pneumonia cases. In particular, all normal images are correctly identified, 58 non-COVID-19 pneumonia images are misclassified as normal, and 1 COVID-19 image is misclassified as normal. Additionally, COVIDNet-CT is capable of distinguishing non-COVID-19 pneumonia cases from COVID-19 cases for the vast majority of these cases. Interestingly, while some COVID-19 cases are incorrectly classified as non-COVID-19 pneumonia cases (113 images), far fewer non-COVID-19 cases are misclassified as COVID-19 cases (13 images).

Based on these results, it is shown that COVIDNet-CT could be used as an effective standalone screening tool for COVID-19 infection, and could also be used effectively in conjunction with RT-PCR testing. However, we note that COVIDNet-CT is trained on images from a single data collection (17), and although this collection is comprised of scans from several institutions, the ability of COVIDNet-CT to generalize to images from other countries, institutions, or CT imaging systems has not been evaluated. As such, COVIDNet-CT could be improved via additional training on a more diverse dataset.

### 3.2. Architecture Comparison

We now compare the performance of the proposed COVIDNet-CT with existing deep neural network architectures for the task of COVID-19 detection from chest CT images. More specifically, we compare it with three state-of-the-art deep neural network architectures: ResNet-50 (25), NASNet-A-Mobile (29), and EfficientNet-B0 (30). In particular, NASNet-A-Mobile and EfficientNet-B0 are deep neural network architectures designed via neural architecture search (NAS) strategies for achieving high architectural and computational efficiency while also achieving high performance.

It can be observed from Table 1 that COVIDNet-CT achieves the highest test accuracy and lowest architectural complexity amongst the tested deep neural network architectures. For example, COVIDNet-CT achieves a test accuracy 0.4% higher than that achieved with the ResNet-50 architecture while having 94.1% fewer parameters and 90.2% fewer FLOPs. Even when compared to the state-of-the-art NASNet-A-Mobile architecture, which was designed using a NAS strategy to achieve a strong balance between accuracy, architectural efficiency, and computational efficiency, the proposed COVIDNet-CT was able to achieve 0.5% higher test accuracy while having 67.4% fewer parameters and 29.6% fewer FLOPs. Lastly, EfficientNet-B0 achieves slightly lower computational complexity than COVIDNet-CT (2.6% reduction in FLOPs), however COVIDNet-CT outperforms EfficientNet-B0 in terms of architectural complexity (65.4% reduction in parameters) and accuracy (0.8% higher test accuracy).

As shown in Tables 2, 5, respectively, COVIDNet-CT achieves higher sensitivity and NPV than the other tested deep neural network architectures across all infection types. Moreover, as shown in Tables 3, 4, respectively, COVIDNet-CT achieves higher specificity and PPV than ResNet-50 and EfficientNet-B0 across all infection types, and also outperforms NASNet-A-Mobile for the non-COVID-19 pneumonia and COVID-19 classes (NASNet-A-Mobile attains higher specificity and PPV for normal control cases). These results highlight the benefits of leveraging machine-driven design exploration to create deep neural network architectures tailored to the task, data, and operational requirements. More specifically, the use of machine-driven design resulted in a network architecture capable of outperforming state-of-the-art architectures across most of the evaluated performance metrics, while also being designed rapidly and automatically. This is particularly relevant in clinical scenarios, as the ability to rapidly build and evaluate new deep neural network architectures is critical in order to adapt to changing data dynamics and operational requirements.

### 3.3. Qualitative Results

**Figure 7:**
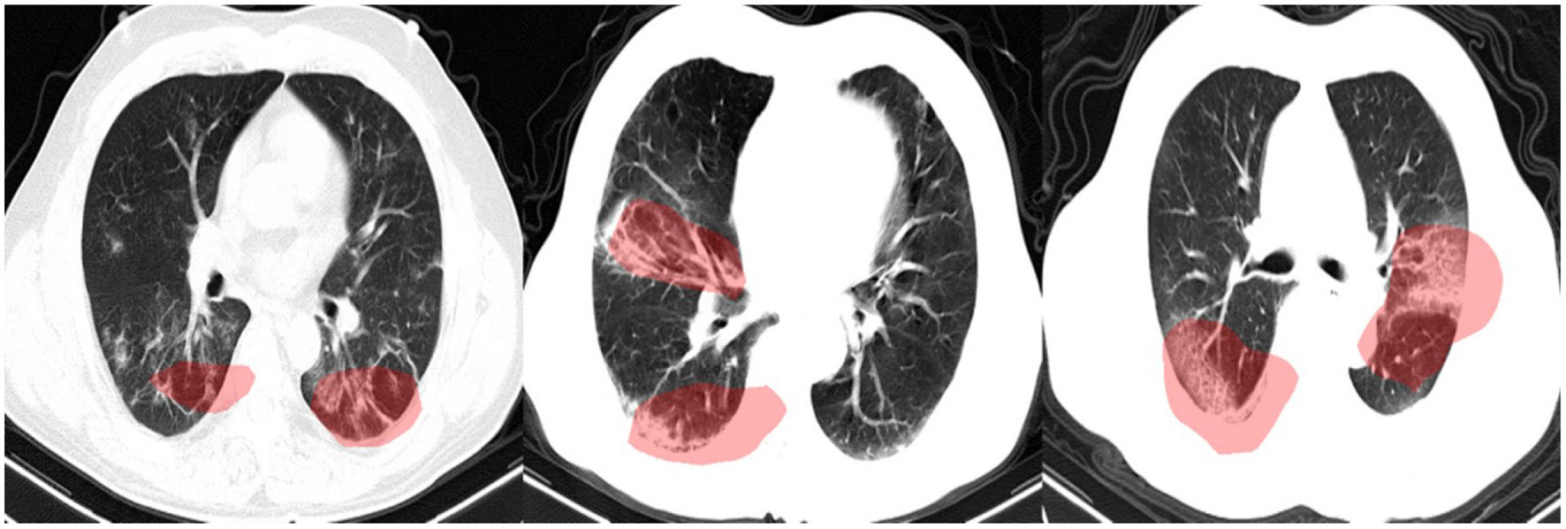
Example chest CT images of COVID-19 cases and their associated critical factors (highlighted in red) as identified by GSInquire (18).

In this study, we leveraged GSInquire (18) to perform explainability-driven performance validation of COVIDNet-CT in order to better understand its decision-making behavior, and to ensure that its decisions are based on diagnostically-relevant imaging features rather than irrelevant visual indicators. Figure 7 shows the critical factors identified by GSInquire in three chest CT images of patients with COVID-19 pneumonia. Examining these visual interpretations, we observe that COVIDNet-CT primarily leverages abnormalities within the lungs in the chest CT images to identify COVID-19 cases, as well as to differentiate these cases from non-COVID-19 pneumonia cases. As previously mentioned, our initial experiments yielded deep neural networks that were found via explainability-driven performance validation to be basing their detection decisions on irrelevant indicators, such as patient tables and imaging artifacts. These findings highlight the importance of leveraging explainability methods when building and evaluating deep neural networks for clinical applications. Furthermore, the ability to interpret how COVIDNet-CT detects COVID-19 cases may help clinicians trust its predictions, and may also help clinicians discover novel visual indicators of COVID-19 infection which could be leveraged in manual screening via CT imaging.

## 4. Discussion

In this study, we introduced COVIDNet-CT, a deep convolutional neural network architecture tailored for detection of COVID-19 cases from chest CT images via machine-driven design exploration. Additionally, we introduced COVIDx-CT, a benchmark CT image dataset consisting of 104,009 chest CT images across 1,489 patients. We quantitatively evaluated COVIDNet-CT using the COVIDx-CT test dataset in terms of accuracy, sensitivity, specificity, PPV, and NPV. Furthermore, we analyzed the predictions of COVIDNet-CT via explainability-driven performance validation to ensure that its predictions are based on relevant image features and to better understand the CT image features associated with COVID-19 infection, which may aid clinicians in CT-based screening. In our analyses, we observed that COVIDNet-CT is highly performant when tested on the COVIDx-CT test dataset, and that abnormalities in the lungs are leveraged by COVIDNet-CT in its decision-making process.

A number of studies have proposed deep learning systems based on chest CT imaging to distinguish COVID-19 cases from non-COVID-19 cases (which may include both normal and abnormal cases) (16, 17, 31–41). Many of the proposed systems further identify non-COVID-19 cases as normal (17, 31, 39, 40), non-COVID-19 pneumonia [e.g., bacterial pneumonia, viral pneumonia, community-acquired pneumonia (CAP), etc.] (17, 31–34, 40, 41), or non-pneumonia (33).

Most of the proposed deep learning systems for CT-based COVID-19 detection make use of pre-existing network architectures which were originally designed for other image analysis tasks. For example, Ardakani et al. (34) compared the performance of 10 existing convolutional neural network (CNN) architectures in distinguishing COVID-19 pneumonia from non-COVID-19 pneumonia, and Jin et al. (38) empirically selected an existing CNN architecture for use in a segmentation-classification system. Additionally, many studies add custom components to pre-existing architectures in order to better tailor them to COVID-19 detection. For example, Xu et al. (31) leveraged a ResNet-18 (24) backbone and added a location-attention classification network to predict COVID-19 probabilities in image patches, which were then used to deduce overall COVID-19 probability. Li et al. (33) and Bai et al. (32) adapted existing 2D CNN architectures to operate on full 3D CT volumes by leveraging pooling operations.

Entirely novel deep neural network architectures have been explored in some studies for COVID-19 case detection. Shah et al. (35) proposed a 10-layer 2D CNN called CTnet-10, but found that it was outperformed by pre-existing architectures. Zheng et al. (37) proposed a 3D CNN called DeCovNet which operates on full 3D CT volumes.

Before COVID-19 detection can occur, many proposed systems require lung and/or lung lesion segmentation (16, 17, 31–33, 36, 37, 39, 40), which necessitates either a segmentation component in the proposed systems or manual segmentation by radiologists. For example, the system proposed by Zhang et al. (17) performs automatic lung lesion segmentation and uses the resulting lung lesion maps as input to a diagnostic network, and the system proposed by Mei et al. (16) requires pre-segmented lung images.

Explainability methods have been leveraged in some studies to investigate the relationship between imaging features and network predictions. Bai et al. (32) and Jin et al. (39) visualized importance variations in chest CT images using Gradient-weighted Class Activation Mapping (Grad-CAM) (42). Similarly, Mei et al. (16) created heatmaps of COVID-19 infection probabilities within receptive fields by upsampling their network's predictions to match chest CT image dimensions. Zhang et al. (17) examined the correlation between key clinical parameters and segmented lung lesion features in chest CT images. Song et al. (40) leveraged a coupled attention module to learn the diagnostic importance of CT image regions.

As discussed above, COVID-19 detection from chest CT images has been investigated extensively in previous studies. However, to the best of the authors' knowledge, the proposed COVIDNet-CT deep neural network architecture is the first to be built using a machine-driven design exploration strategy specifically for COVID-19 detection from chest CT images. Moreover, this is the first study to perform an explainability-driven performance validation using an explainability method geared toward identifying specific critical factors in chest CT images. This is in contrast to the more general heatmap-based or bounding-box-based explainability methods leveraged in previous studies, as these methods illustrate importance variations within images without identifying specific critical factors.

While the proposed COVIDNet-CT is not yet suitable for clinical use, we publicly released COVIDNet-CT and instructions for constructing the COVIDx-CT dataset as part of the COVID-Net open intiative in order to encourage broad usage and improvement by the research community. In particular, for COVIDNet-CT to be considered for clinical use, clinical studies examining its generalization capabilities and stability would be required, as well as an exploration of how exactly it distinguishes COVID-19 pneumonia cases from other pneumonia cases. These steps are crucial since performance, reliability, and transparency are of paramount importance in clinical applications. In the future, the performance and generalizability of COVIDNet-CT may be improved by expanding and diversifying the COVIDx-CT dataset, and COVIDNet-CT may also be extended to additional clinical tasks, such as mortality risk stratification, lung function analysis, COVID-19 case triaging, and treatment planning. However, the ability to build solutions for these tasks is contingent on the availability of high-quality datasets. Finally, additional analysis of the explainability results may be performed in the future to identify key patterns in the CT images which may aid clinicians in manual screening.

## Data Availability Statement

The data analyzed for this study can be found in the CNCB repository: http://ncov-ai.big.ac.cn/download?lang=en. Instructions for generating the COVIDx-CT dataset can be found in the COVIDNet-CT repository: https://github.com/haydengunraj/COVIDNet-CT.

## Ethics Statement

The studies involving human participants were reviewed and approved by the University of Waterloo Ethics Board. Written informed consent from the participants' legal guardian/next of kin was not required to participate in this study in accordance with the national legislation and the institutional requirements.

## Author Contributions

HG and AW conceived the study and analyzed the results. HG, LW, and AW conducted the experiments. All authors reviewed the manuscript.

## Conflict of Interest

LW and AW are affiliated with DarwinAI Corp. DarwinAI Corp., NVIDIA Corp., and Hewlett Packard Enterprise Co. have provided computing support for this work. In particular, DarwinAI Corp. provided access to their deep learning development platform, and NVIDIA Corp. and Hewlett Packard Enterprise Co. provided access to GPU computing resources. The remaining author declares that the research was conducted in the absence of any commercial or financial relationships that could be construed as a potential conflict of interest.

## Footnotes

^1^https://github.com/haydengunraj/COVIDNet-CT
